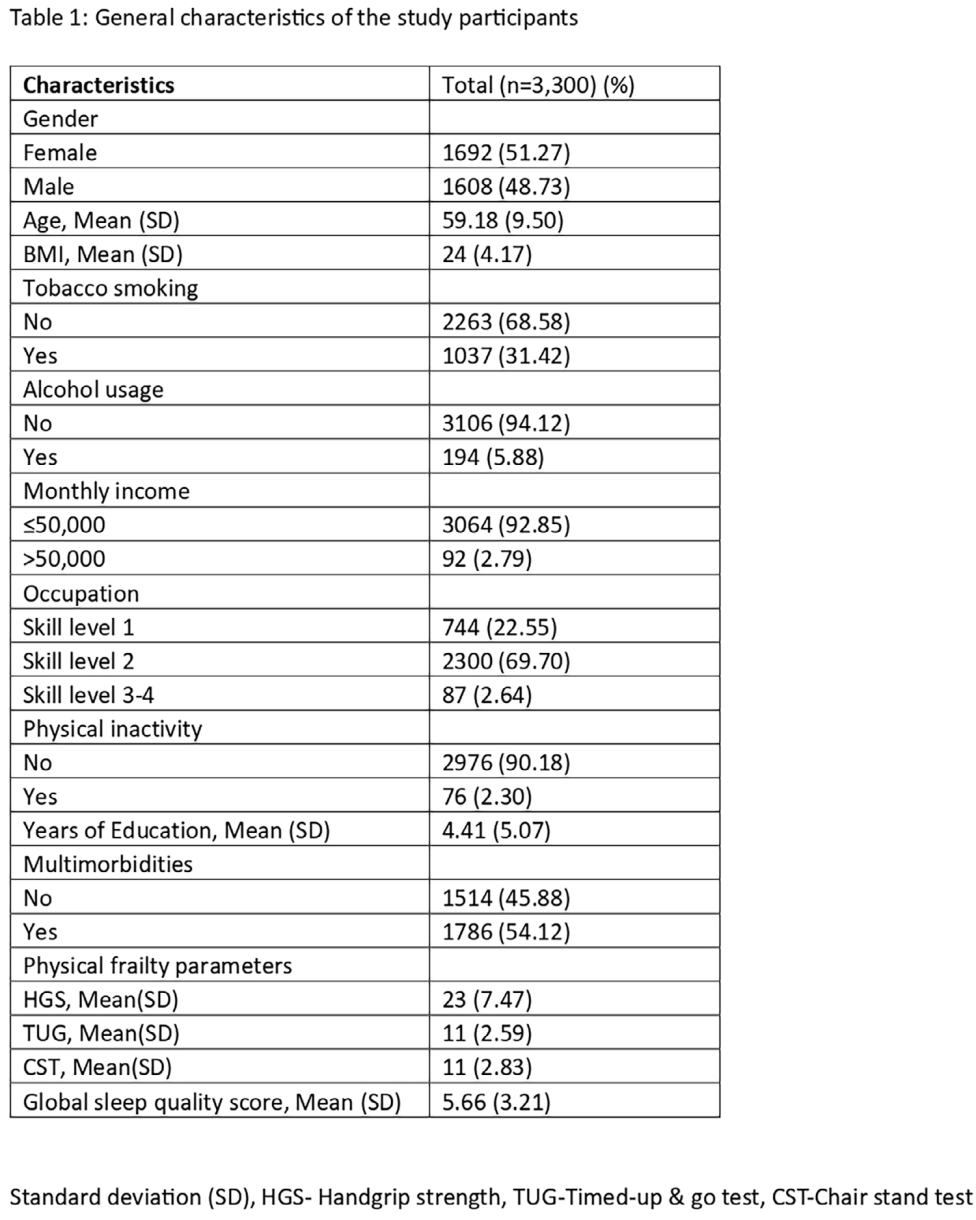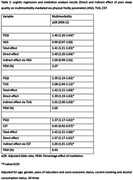# Impact of physical frailty parameters on the association between sleep quality and multimorbidity

**DOI:** 10.1002/alz.091285

**Published:** 2025-01-09

**Authors:** Priya Chatterjee, Pravin Sahadevan, Hitesh Pradhan, Jonas S. Sundarakumar

**Affiliations:** ^1^ Centre for Brain Research, Indian Institute of Science, Bangalore, Karnataka India

## Abstract

**Background:**

Previous studies have reported that poor sleep quality is associated with several adverse outcomes including multimorbidity, particularly in the aging population. Another common occurrence in the elderly is frailty. We aim to study the mediating effect of objective physical frailty parameters on the association between sleep quality and multimorbidity in an aging rural Indian population.

**Method:**

We used baseline data (n = 3300) from the ongoing Srinivaspura Aging Neuro Senescence and Cognition (SANSCOG) cohort study, conducted in the rural areas of the state of Karnataka in India. Objective physical frailty parameters included in the study were hand grip strength (HGS), chair stand test (CST), timed up and go (TUG) tests. Pittsburgh Sleep Quality Index (PSQI) was used to assess sleep quality. Multimorbidity was categorized based on the presence of two or more diagnosed chronic disease conditions (self‐reported/objective measures). Multivariable logistic regression was performed to find out the association between poor sleep quality and multimorbidity. Mediation analyses [using Karlson–Holm–Breen (KHB) method] were performed separately for each of the physical frailty parameters on the association between poor sleep quality and multimorbidity and results were reported as adjusted Odds ratio (a‐OR) and 95% CI. Models were adjusted for age, gender, education, socioeconomic status, current smoking and alcohol consumption status.

**Result:**

A total of 39.09% participants had poor sleep quality (PSQI>5) and 54.12% had multimorbidity. Participants with poor sleep quality had higher odds of being multimorbid [a‐OR: 1.41, 95% CI: 1.21‐1.65]. Among the physical frailty parameters, individuals with higher TUG time had higher odds of having multimorbidity [a‐OR: 1.08, 95% CI: 1.04‐1.11]. People with higher score in the CST had lower odds of being multimorbid [aOR: 0.95, 95% CI: 0.92‐0.97]. Performance in TUG and CST significantly mediated the association between poor sleep quality and multimorbidity, although the mediated effects were small (TUG‐ 5.00% and CST‐ 6.83%). However, no association was found between HGS and multimorbidity.

**Conclusion:**

Our study finds that poor sleep quality is associated with increased risk of multimorbidity and that poor performance in physical frailty parameters mediates this association.